# Risky sexual behaviors and associated factors among adolescent in Gedeo Zone, South Ethiopia: a community based cross-sectional study

**DOI:** 10.1038/s41598-024-67944-4

**Published:** 2024-08-28

**Authors:** Yohannes Addisu Wondimagegne, Adane Tesfaye Anbese

**Affiliations:** College of Health Science and Medicine, School of Public Health, Dilla University, Dilla, Ethiopia

**Keywords:** Adolescent, Risky sexual behaviors, Gedeo Zone, South Ethiopia, Health care, Risk factors

## Abstract

Adolescents represent 16% of the global population and they are identified as a critical demographic group for promoting sexual health. Adolescents are susceptible to engaging in risky sexual behaviors (RSB) such as early sexual initiation, having multiple sexual partners, substance use during sexual encounters and practicing unsafe sex. Adolescents represent 16% of the global population and they are identified as a critical demographic group for promoting sexual health. Adolescents are susceptible to engaging in risky sexual behaviors (RSB) such as early sexual initiation, having multiple sexual partners, substance use during sexual encounters and practicing unsafe sex. To assess risky Sexual behaviors and associated factors among adolescent in Gedeo Zone, Southern Ethiopia: A community based cross-sectional study. A community based cross-sectional study was conducted in Gedeo Zone among adolescents. A total of 2780 (99.3%) adolescents were participated in the study and gave the response rate of 99.3%. A pre-tested structured questionnaire was used to gather the data and analyzed by using SPSS version 23. During analysis initially bivariable logistic regression model was used then, those variables with a level of significant at a *P*-value ≤ 0.25 were considered as candidate for multivariable logistic regression model. A level of significant at a *P*-value ≤ 0.05 was considered as statistically significant in this study. Out of 428 sexually active adolescent 334 (78%) exposed to risky sexual practice. More than half 54.3% of adolescent was protestant in religion followed by Orthodox 34.2% and Muslim 11.5%. In terms of ethnicity, Gedeo 67.4% was the dominant ethnic group in the study area. Mean age at sexual initiation was 15 ± 1.8.Residence AOR 1.14 (1.36–5.25), Sex AOR 2.77 (1.31–5.86), Age AOR 2.01 (1.41–6.39), School attending AOR 1.93 (1.27–5.75), Watching Pornographies AOR 2.51 (1.36–4.62) and Parental monitoring AOR 2.10 (1.07–4.10) were independent predictor of risky sexual practice in this study. The prevalence of risky sexual behavior was found to be alarming among adolescents aged 14–19 years, mostly rural and female adolescents and those adolescent start sexual practice earlier exposed to risky sexual practice than their counter parts. Sexual urge, watching pornography and not attending school were the major factor for risky sexual behaviors of adolescent**.** Parental over all control can protect risky sexual behaviors among adolescent.

## Introduction

According to the World Health Organization (WHO), adolescents represent 16% of the global population and they are identified as a critical demographic group for promoting sexual health^1^. Adolescents are susceptible to engaging in risky sexual behaviors (RSB) such as early sexual initiation, having multiple sexual partners, substance use during sexual encounters and practicing unsafe sex. These practices are thought to be a serious public health concern because they raise the risk of STIs and unintended pregnancy^2,3^.

Recent systematic reviews and meta-analyses have reported that parental monitoring is associated with delayed sexual initiation, greater condom and contraceptive use^4–6^. Although these complex interplays illustrate the pivotal role parents can play in influencing adolescent sexual behavior^7,8^*.*

Addictive substances such as khat’ leaves (Catha Edulis—a green plant that grows in Yemen and East Africa), tobacco and alcohol are widely used by adolescents which can be a potential cause for RSB (5). Generally, RSB is common among adolescents in Sub Saharan Africa^9,10^. Studies on adolescents´ sexual behaviors are important because promoting healthy behaviors and preventing risky sex among adolescents are important for country´s future health^1^ and the attainment of Sustainable Development Goals (SDG) of 2030^11^ .

In particular, research on the Human immunodeficiency virus/ acquired immune deficiency syndrome (HIV/AIDS ) epidemic established that sub-Saharan Africa (SSA) accounts for 60% of all people living with HIV/AIDS due to the RSB, which has hampered the quality of life^12^.According to the Ethiopian Demographic and Health Survey (EDHS), the country is characterized by adolescent population contributing 24.4% of the total population^13^.

In Ethiopian, among sexually active adolescents 48.5% had multiple sexual partners and 66% did not use condom consistently^14–16^. moreover, 44% of pregnancies among adolescents were unintended of which 46% ended in abortion^17^. The Ethiopian government has designed and executed several strategies to improve the SRH of adolescents through the provision of SRH services to youth^18^. The main aim of the current study was to assess risky sexual behaviors and associated factors among adolescent in Gedeo Zone, Southern Ethiopia.

## Method

### Study area

Community based cross-sectional study was conducted in rural and urban areas of Gedeo Zone.

The Zone has four towns, namely, Dilla and Yirgachafe, wenago and Gedeb and nine districts. Districts of Gedeo Zone are Dilla Zurea, Yirgachafe Zurea, Bule, Gedeb , Kochere, Wenago, Cheleleketu, Raphe and Chorso.

### Study design and period

A Community-based cross-sectional study design was applied and the study was conducted from June 2, 2021 to June 30, 2021.

### Source and study population

Those adolescent who reside in Gedeo Zone age 14–19 was considered as source population, whereas study population included randomly chosen adolescent age 14–19 during the data collection period.

### Inclusion and exclusion criteria

Adolescents who were randomly selected in Gedeo Zone were included in this study. Moreover, adolescents were eligible if they provided written and informed consent. Nevertheless, the study population who declined to offer informed & written consent, who was critically ill during the data collection period, and who declined to respond question by data collectors were excluded from the study.

### Sampling size determination

Sample size was calculated using Epi-info version 7 with the assumption of 14.7% prevalence of risky sexual behavior among unexposed, power of 80%, ratio of unexposed to expose of 1 and assuming 20.7% prevalence among exposed group^16,19^**.** Accordingly, the calculated sample became 1272. Considering design effect of 2 and 10% non-response rate, the final sample became 2798. However, from this sample size, merely 428 had exposure to sexual practice in the last six months and became the study population.

### Sampling procedure

Study area was stratified into urban and rural area. Then, multi-stage sampling techniques was used initially, then three rural districts were randomly selected out of nine existing rural districts and two urban administrative were randomly assigned from three urban districts. After identification of sub-districts, Census was conducted to have a sampling frame. A stratified sampling approach was used to select study participants from the selected sub-districts. To determine the number of participants in each sub-district, a proportional allocation for population size was used. Finally study participants were chosen by systematic sampling technique.

### Data collection tools and procedures

A validated questionnaire modified slightly from the Ethiopia Demographic and Health Survey (EDHS 2016) & earlier related publications^20,21^. The questionnaire mainly consists of closed-ended questions with a few open-ended ones. Before the data collection, pretesting was conducted in the adjacent region of the study area. For simplicity of use, the questionnaire was prepared in English, interpreted into Amharic, and then returned back to English to guarantee reliability. Fifteen data collector and five BSc midwives employed to collect and supervise the data collection process respectively. A three-day training session on the study's goals, data collection procedure, daily checks for questionnaire completion, and data quality were given to field workers. The overall processes were under the control of the principal investigator. Items included in the analysis with a cronbach 's alpha of 0.7 or higher were applied to confirm the reliability of the instrument.

### Operational definition

#### Sexually activity

These are those adolescents age 13–19 having a history of ever vaginal sexual intercourse^21^.

#### Currently sexually active

These are those adolescents age 13–19 having a history of last six-month vaginal sexual intercourse^22^.

#### Wealth index

Household's assets that are durable and semi-durable goods were used to describe household economic status. Household questionnaire that was used by Ethiopian demographic and health survey to measure household's socioeconomic status were applied to assess the wealth index. The wealth index was calculated by principal component analysis using data of household's assets then the respondents' families were classified into three according to wealth status and classified as low, medium and high^23^.

#### Risky sexual behaviour

It was defined as unprotected sex (inconsistent use of condoms), having multiple sexual partner, starting sex before age of 18 years and sex with commercial sex workers^24^.

#### Parental monitoring

Parental monitoring was assessed using a six-item Silverberg’s parental monitoring scale. Items were scored from 1 (never) to 5 (always). Those who scored lower than the median was considered as no Parental monitoring and who score above the median are considered as have parental monitoring^25^.

#### Parental communication

It was measured with a five-item parent-adolescent communication scale. Items were scored from 1 (never) to 4 (often). Those who scored lower than the median value was considered as low parental communication and above the median as high parental communication during the last six months^21^.

### Data analysis

The data template format was prepared in Epi data version 3.1 for data entry then data were exported to SPSS version 24, categorized and sorted to facilitate its analysis. Before analyses, the data were cleaned, and the distributions and multicollinearity of the variables were checked. Complete case analyses were carried out for missing data. A descriptive analysis of the background characteristics was performed. Normality was checked for continuous variables. Bivariate logistic regression analysis was used to test possible association of the independent variables with the dependent one a *P*-value less than 0.25 in bivariate logistic regression were entered into multivariate logistic regression. Furthermore, multivariate logistic regression analysis was used to see the net effects of each of the independent variables in explaining variation in the outcome variables. A *P*-value ≤ 0.05 was considered as statistically significant association. Hosmer Lemeshow was used in the analyses to check the model's goodness of fit.

### Ethical issues

Ethical clearance was obtained from Dilla University, College of Health Sciences and Medicine Ethical Review board (IRB). Informed consent was obtained from the parent of study participants prior to interview and purpose of the study was explained deeply in the consent form for the respondents and their parents. Confidentiality of the information obtained was assured and privacy of the respondents was maintained. All methods were carried out in accordance with relevant guidelines and regulations or declaration of Helsinki^26^.

## Results

Out of 428 sexually active adolescent 334 (78%) exposed to risky sexual practice. More than half 54.3% of adolescent was protestant in religion followed by Orthodox 34.2% and Muslim 11.5%. In terms of ethnicity, Gedeo 67.4% was the dominant ethnic group in the study area (Table 1**)**.

**Table 1:** Socio-demographic characteristics of study participant in Gedeo Zone, South Ethiopia, 2021.

Characteristics	Category	Frequency	Percentage
Age (Year)	14–17	322	75.2
	18–19	106	24.8
	Mean age (± SD) (15 ± 1.8.)		
Residence	Urban	154	35.9
	Rural	274	64.1
Sex	Female	230	53.8
	Male	198	46.2
Occupation	Student	300	70.1
	Jobless	42	9.9
	Student and work	22	5.2
	Merchant	38	8.8
	Other	26	6.0
Educational status	Not literate	41	9.5
	Elementary	166	38.8
	Secondary or above	221	51.7
Wealth index	Low	153	35.7
	Medium	114	26.7
	High	161	37.6

### Type of risky sexual behavior adolescent experience

**Figure 1:**
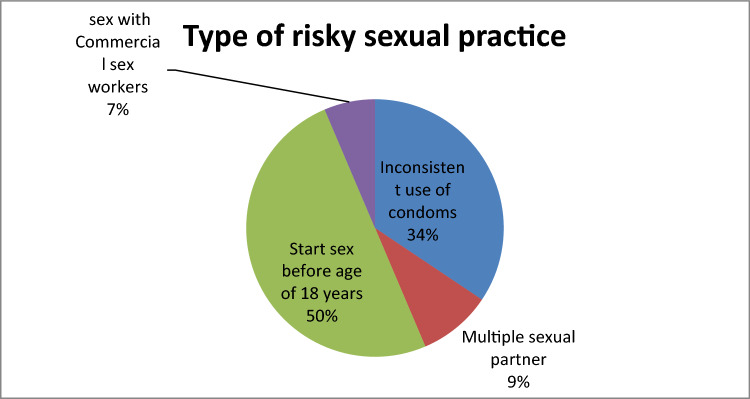
Type of risky sexual behavior adolescent experience in Gedeo Zone, South Ethiopia, 2021.

In the current study a totally of 334 (78%) study participants were sexually at risk, out of this number, 115 (34.4%) exposed to unprotected sex (inconsistent use of condoms), whereas 31 (9.2%) had multiple sexual partner, the rest 167 (50%) and 21 (6.4%) start sex before age of 18 years and had sex with commercial sex workers respectively (Fig. 1**).**

### Factors associated with risky sexual behavior

In bivariate logistic regression, place of residence, Sex, Age, School attending, Wealth index , Khat Chewing, Alcohol Taking, Ever screened for HIV/AIDs, Parents use substance, Father Educational Status and having friends who use substance, Watching Pornographies, Parental monitoring were associated with risky Sexual behavior at p value less than 0.25. However, in multivariate logistic regression, place of residence, Sex, Age, School attending, Watching Pornographies, and Parental monitoring were associated with risky Sexual behavior at p value less than **0.25.**

However, in multivariate logistic regression, residence, Sex, Age, School attending, Watching Pornographies, Parental monitoring was found to be statistically associated with risky Sexual behavior. Adolescent who reside in rural area were 1.1 times more likely exposed to risky sexual practice than adolescent who reside in urban area (AOR = 1.14; CI: 1.36–5.25). On the other hand, female adolescents were 2.7 times more likely exposed to risky sexual practice compared to their male counter parts (AOR = 2.7; 95% CI: 1.31–5.86), adolescent age 14–17 year were 2 times more likely exposed to risky sexual practice compared to adolescent age 18–19 counter parts (AOR = 2.0; 95% CI: 1.41–6.39).

Those adolescent did not attending school during the study period were 1.9 times more likely exposed to risky sexual practice compared to adolescent that attended school (AOR = 2.9; 95% CI: 1.27–7.75), Those adolescent watching Pornographies were 2.5 times more likely exposed to risky sexual practice compared to those adolescent who never watching Pornographies AOR 2.51 (1.36–4.62), and finally those adolescent who did not experience Parental monitoring were 2.10 times more likely exposed to risky sexual practice compared to their counter parts (AOR = 2.1; 95% CI: (1.07–4.10) **(**Table 2**).**

**Table 2:** Factors associated with risky sexual behavior among adolescent in Gedeo Zone, South Ethiopia, 2021.

Characteristics	Risky Behavior		COR(95%CI)	AOR(95%CI)	P value
Residence	Yes	No			
Urban	130(83.3)	24(16.7)	1	Ref	1
Rural	204(70.4)	70(29.6)	1.85 (1.03–4.28)	1.14 (1.36–5.25)	0.007
Sex					
Female	202(87.8)	28(12.2)	3.60 (1.62–5.75)	2.77 (1.31–5.86)	0.008
Male	132(66.7)	66(33.3)	1	Ref	1
Age (Year)					
14–17	266(82.6)	56(17.4)	2.65 (1.49–5.43)	2.01 (1.41–6.39)	0.004
18–19	68(64.1)	38(35.9)	1	Ref	1
School attending					
Yes	232(80.0)	58(20.0)	1	Ref	1
No	102(64.6)	56(35.4)	2.24 (1.18–4.06)	1.93 (1.27–5.75)	0.012
Watching pornographies					
Yes	201(90.1)	22 (9.1)	4.9 (1.90–5.74)	2.51 (1.36–4.62)	0.003
No	133 (64.8)	72 (35.2)	1	Ref	1
Parental monitoring					
Yes	88(64.7)	48(35.3)	1	Ref	1
No	246(84.2)	46(15.8)	2.9 (1.98–4.36)	2.10 (1.07–4.10)	0.029
Parental communication					
Yes	134(71.27)	54(28.73)	1	Ref	1
No	200(83.3)	40 (16.7)	2.01 (1.21–3.93)	1.48 (0.81–2.72)	0.197

## Discussion

The present studies try to explore risky sexual behavior and associated factors among adolescent in Gedeo Zone. In the current study, 428 adolescent were currently sexually active. In the current study a totally of 334 (78%) study participants were sexually at risk, out of this number, 115 (34.4%) exposed to unprotected sex (inconsistent use of condoms), whereas 31 (9.2%) had multiple sexual partner, the rest 167 (50%) and 21 (6.4%) start sex before age of 18 years and had sex with commercial sex workers respectively.

In the current study, 428 (15.2%) of adolescents had ever practiced sexual intercourse in their life time. This was lower (12%) than study conducted in northern Ethiopia^27^, The reason for this discrepancy may arise difference in socio-demographic variation and other related factors specific to the study setting.

The current study showed that those adolescents who reside in rural areas were two times more likely involved in risky sexual practice as compared to their urban counter part this finding was similar with a study conducted in Nigeria^28^ the main reason may be in rural areas adolescent exposed to unprotected sexual practice as a result of low access to SRH education and there is very limited Youth friendly service targeting adolescent SRH service in rural area as compared to urban area^11^.

In the current study those female adolescent were more likely exposed to risky sexual practice than male adolescent this finding was similar with a study conducted in west Ethiopia^29^. the reason may be female adolescent face many pressure from their peer and other adult male to exposed to sexual practice which potentially leads to risky sexual behavior moreover female adolescent are more likely exposed to gender based violence that may exposed them to risky sexual practice.

In the current study low exposure to pornographic material and Parental monitoring increase risky sexual practice which is supported by other studies^30^. The reason might be exposure to pornography leads to sexual provocation, impulsiveness and increase possibility to engaged into risky sexual practice^19,27^. Similarly, study from West Ethiopia Gudru district showed that those adolescent who did not have parental monitoring were more likely exposed to risky sexual practice^29^.

### Limitation of the study

There is social desirability bias like under reporting of sexual practice and number of sexual partners especially among female and over reporting among male adolescent, in addition the study participant may face a recall bias.

## Conclusion

The prevalence of risky sexual behavior was found to be alarming among adolescents aged 14–19 years, mostly rural and female adolescents and those adolescent start sexual practice earlier exposed to risky sexual practice than their counter parts. Sexual urge, watching pornography and not attending school were the major factor for risky sexual behaviors of adolescent**.** Parental over all control can protect risky sexual behaviors among adolescent.

## Data Availability

Data availability statement Data are available upon reasonable request from the corresponding author: yohannes_24@yahoo.com.
